# Supplementary material for: Renalase Overexpression-Mediated Excessive Metabolism of Peripheral Dopamine, DOPAL Accumulation, and α-Synuclein Aggregation in Baroreflex Afferents Contribute to Neuronal Degeneration and Autonomic Dysfunction
Source: Biomedicines. 2025 May 20;13(5):1243. doi: 10.3390/biomedicines13051243 (PMC12109403; doi:10.3390/biomedicines13051243)
Supplement: Supplementary file 1 [file biomedicines-13-01243-s001.zip › biomedicines-3571570-supplementary.pdf]

biomedicines

MDPI

Online only supplemental data

Renalase Overexpression-Mediated Excessive Metabolism of Peripheral Dopamine, DOPAL Accumulation, and α-Synuclein Aggregation in Baroreflex Afferents Contribute to Neuronal Degeneration and Autonomic Dysfunction

Xue Xiong †, Yin-Zhi Xu †, Yan Zhang, Hong-Fei Zhang, Tian-Min Dou, Xing-Yu Li, Zhao-Yuan Xu, Chang-Peng Cui, Xue-Lian Li and Bai-Yan Li *

State Key Laboratory of Frigid Zone Cardiovascular Diseases (SKLFZCD), Department of Pharmacology (State Key Laboratory-Province Key Laboratories of Biomedicine-Pharmaceutics of China, Key Laboratory of Cardiovascular Research, Ministry of Education), College of Pharmacy, Harbin Medical University, Harbin 150086, China; xuexiong@buffalo.edu (X.X.); xu2019@purdue.edu (Y.-Z.X.); zhangy0393@163.com (Y.Z.);15145227962@163.com (H.-F.Z.); 18712028460@163.com (T.-M.D.); 15097657976@163.com (X.-Y.L.); xu1990@purdue.edu (Z.-Y.X.); 18845146616@163.com (C.-P.C.); lixuelian@hrbmu.edu.cn (X.-L.L.)

* Correspondence: liby@ems.hrbmu.edu.cn; Tel.: +86-(0451)-8667-1354

† These authors contributed equally to this work.

Table S1. All primers used in the experiments are listed.

Primers	Sequence
β-actin	Fwd: 5'-TCTTCCAGCCTTCCTTCCTG-3'
	Rev: 5'-CACACAGAGTACTTGCGCTC-3'
α-Syn	Fwd: 5'-GAGGGAGTCGTTCATGGAGT-3'
	Rev: 5'-CATTTGTCACTTGCTCTTTGG-3'
TH	Fwd: 5'-CTACTGTCCGCCCGTGATTT-3'
	Rev: 5'-GGTCAGCCAACATGGGTACA-3'
DYN	Fwd: 5'-AGCTTTGGCACATCAGGAGT-3'
	Rev: 5'-CAGCAGATAGCAAGCTCACGA-3'
Renalase	Fwd: 5'-AAGAAGCGCAACACAGAGT-3'
	Rev: 5'-TGTGACTCCAAATGGGACGGT-3'
ALDH	Fwd: 5'-CGTCACCAGCAAAGTGTTGT-3'
	Rev: 5'-AGACAGGGAGCTACGACAGA-3'
KIF	Fwd: 5'-ACCGACACACATTGCAGGT-3'
	Rev: 5'-CAGCTGTACAAGCCAGGAGT-3'

Table S2. Behavioral analysis of Parkinson's rats after intraperitoneal injection of dopamine receptor agonist apomorphine. A body rotation of more than 7/min is considered a successful establishment of the model.

No. Rat	1 w	2 w	3 w	4 w	5 w	6 w
**1-1**	0	3	6	**8**	**8**	**12**
**1-3**	2	1	4	7	**9**	**8**
**1-4**	3	0	3	**9**	**10**	**11**
**1-5**	2	6	**8**	**10**	**10**	**11**
**2-1**	0	1	4	**10**	**13**	**14**
**2-2**	1	2	5	**12**	**10**	**13**
**2-5**	2	4	6	7	**10**	**10**
**3-3**	3	5	4	6	**11**	**9**
**3-5**	4	6	**9**	**10**	**9**	**8**
**4-3**	6	**8**	**10**	**11**	**10**	**12**
**Mean**	**2.556**	**3.667**	**5.889**	**9.111**	**10.222**	**10.667**
**SD**	**1.641**	**2.625**	**2.378**	**1.912**	**1.133**	**2.000**
**%**	**0%**	**10%**	**30%**	**70%**	**100%**	**100%**

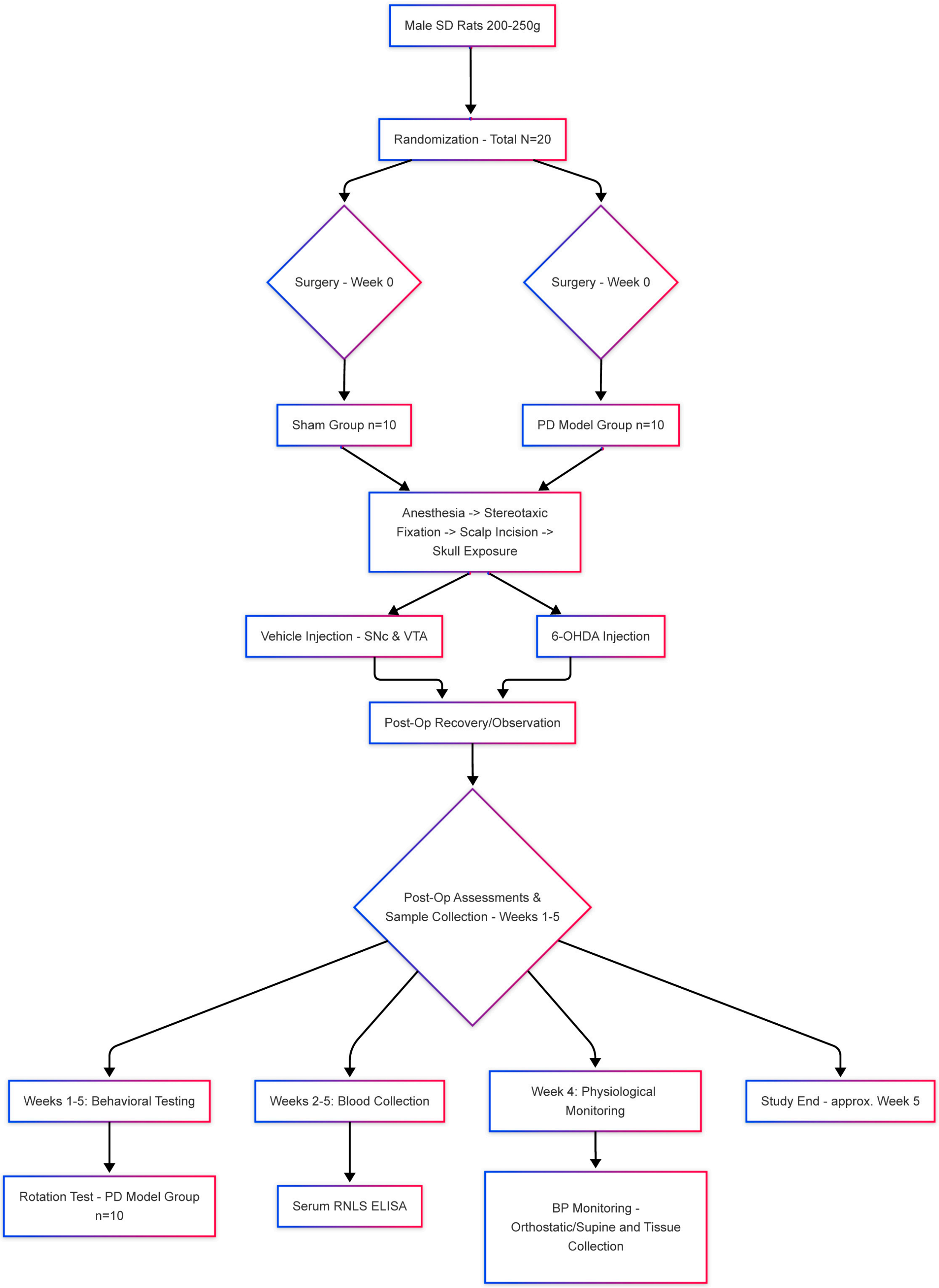

Figure S1. Rats experiment scheme.

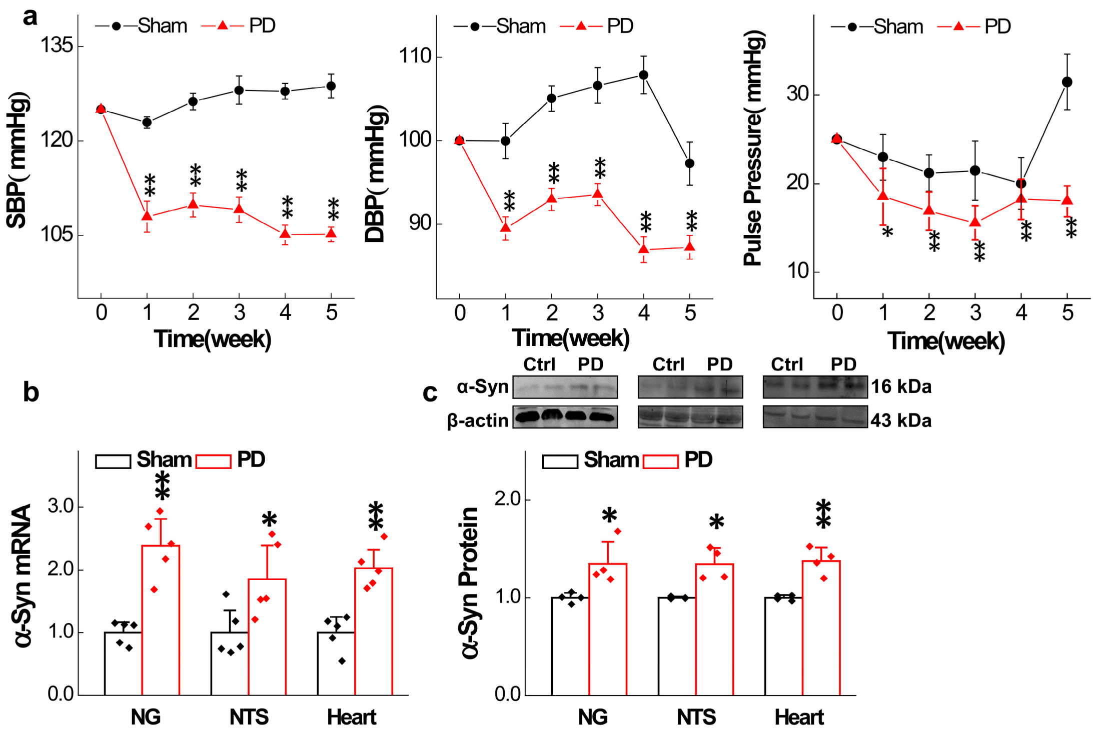

Figure S2. Changes in blood pressure and the content of α-Syn in vivo increased after 6-OHDA brain injection in rats. **a** Shows the systolic blood pressure (SBP), diastolic blood pressure (DBP), and pulse pressure difference, respectively (n = 7 rats per group). **b** qRT-PCR detection of α-Syn expression in NG, NTS and heart (4th week after 6-OHDA application) (n = 5 rats per group). **c** Western blot analysis showing α-Syn protein level in NG, NTS and heart (n = 4 rats per group). Data were presented as mean ± SD, * $p < 0.05$ and ** $p < 0.01$ vs. Sham.
